# Unexplained Neonatal Cardiorespiratory Collapse at Five Minutes of Age

**DOI:** 10.1155/2016/7206572

**Published:** 2016-02-24

**Authors:** Sona Zaleta, Sarah Miller, Prashant Kumar

**Affiliations:** Department of Paediatrics, Timaru Public Hospital, Timaru 7910, New Zealand

## Abstract

We report a case in which a term neonate suffered cardiorespiratory collapse at five minutes of age following an uncomplicated delivery and Apgar score of eight at one minute. Following prolonged cardiopulmonary resuscitation, the infant recovered well with no neurological deficit. Although sudden and unexpected postnatal collapse has been extensively described, this case does not fulfil its definition criteria. It provides a diagnostic challenge for clinicians and to the best of our knowledge is the first report of unexplained cardiorespiratory collapse at five minutes of age. The case serves as a timely reminder that cord gas analysis is recommended in all cases of potential fetal compromise and that Apgar scores should be used with caution as a predictor of neurological sequelae.

## 1. Introduction

Apgar scores assess the clinical state and physiological transition of a newborn infant [1]. Typically scores improve between one, five, and ten minutes of age [1]. Cases in which Apgar scores decline are rare. We report a challenging case in which a term neonate, with a one-minute Apgar score of eight, experienced a rapid, unexpected cardiorespiratory collapse at five minutes of age. Prolonged resuscitation resulted in a successful outcome whereby at sixteen weeks the patient has no neurological complications. Although sudden and unexpected postnatal collapse (SUPC) has been previously described, this case does not fulfil its definition criteria. To our knowledge, it is the first reported case of unexplained cardiorespiratory collapse as early as five minutes of age.

## 2. Case Presentation

A 2700 g female was born at term to a fit and well 38-year-old primigravida mother following an uncomplicated in vitro fertilisation (IVF) pregnancy. Ventouse delivery was performed in the operating theatre due to suboptimal progress in the second stage of labour and a nonreassuring cardiotocography (CTG) trace. Policy at our hospital dictates that there must be a paediatric consultant present at every emergency delivery, together with a consultant anaesthetist and obstetrician.

Whilst delivery was uncomplicated, fresh meconium was noted following delivery of the head. One-minute Apgar score was eight. At three minutes, intramuscular vitamin K was given. The patient was pink and moving spontaneously but intermittently grunting. Continuous positive airways pressure (CPAP) with room air was therefore initiated. At five minutes she became white with no respiratory effort and absent heart sounds. Immediate resuscitation was commenced by the paediatric consultant and accompanying team, including intubation, cardiac compression, and administration of three doses of adrenaline via an umbilical venous catheter. There was no evidence of meconium at intubation. Chest rise and breath sounds were noted following intubation with assisted ventilation, and expiratory carbon dioxide was confirmed via a colorimetric end-tidal carbon dioxide monitor. Intermittent positive pressure ventilation (IPPV) with 100% oxygen was initiated. Further help, in the form of an additional experienced paediatric consultant and senior paediatric nurse practitioner, was summoned during the resuscitation.

**Figure 1:**
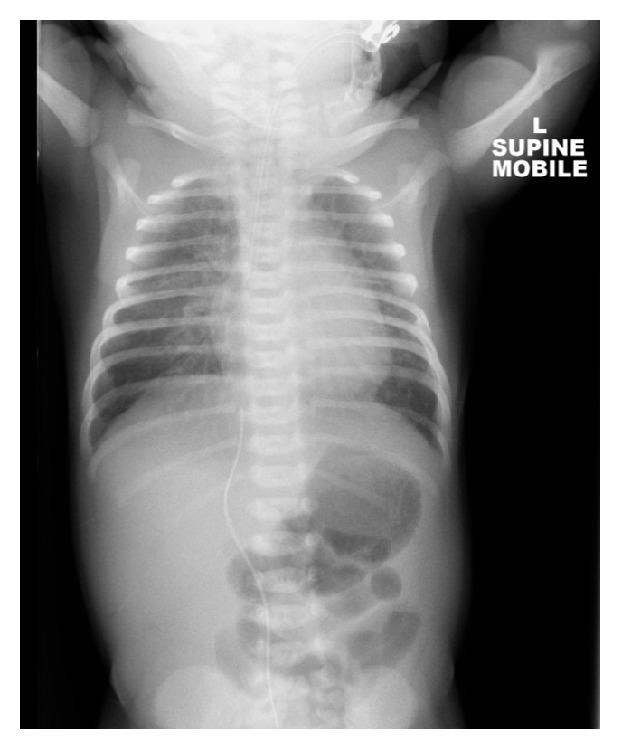
Chest X-ray and abdominal X-ray at two hours of age. The endotracheal tube tip is in the right main bronchus and was withdrawn 15–20 mm. The cardiothalamic silhouette is normal and lung and pleural spaces are clear. No pneumothorax is seen. The umbilical venous catheter lies to the right of the midline at T10.

Cardiac compression continued for 15 minutes until return of heart beat. Five minutes following this, the patient began gasping and respirations normalised. Oxygen saturations improved to 100%. CPAP via endotracheal tube was commenced and inspired oxygen weaned to room air. Cardiovascular examination was normal with no abnormalities seen on cardiac monitoring. Bloods demonstrated haemoglobin 129 g/L, white count 23.8 × 10^9^/L, platelets 311 × 10^9^/L, sodium 136 mmol/L, potassium 2.8 mmol/L, urea 5.2 mmol/L, creatinine 128 *μ*mol/L, and glucose 12.8 mmol/L. Liver function tests, magnesium, phosphate, calcium, and c-reactive protein were unremarkable. No cord gas had been taken. Initial umbilical venous blood gas at 90 minutes of age demonstrated a pH 7.03, pCO2 28.8 mmHg, pO2 55.9 mmHg, HCO3 7.5 mmol/L, BE −23.2 mmol/L, and lactate 16.19 mmol/L. The profound metabolic acidosis normalised over 24 hours with supportive management only. IV antibiotics were administered and continued for 24 hours, however blood cultures were negative and chest X-ray showed no collapse or consolidation (Figure 1). Due to a rapid recovery, cerebral spinal fluid was not obtained.

**Figure 2:**
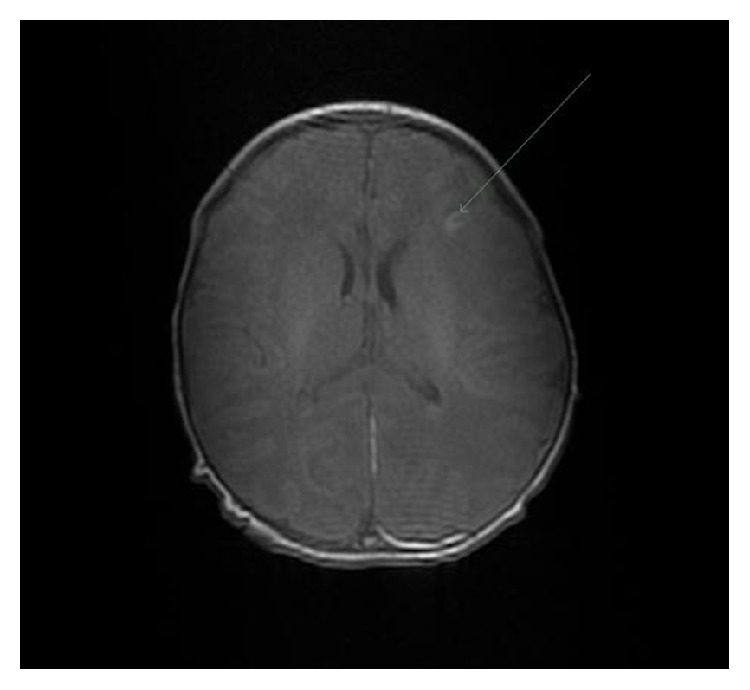
Magnetic resonance imaging demonstrating a 1–1.5 cm focal area of parenchymal abnormality in the left frontal lobe anterolaterally, appearances of which are consistent with infarction.

By 150 minutes, the patient was displaying spontaneous limb movements, normal grasp and gag reflexes, symmetrical eye movements, and good tone. At four hours she was successfully extubated. Head ultrasound at 24 hours detected no abnormality. She did not receive head cooling. Over subsequent days, she exhibited normal behaviour and established feeding well. There were no signs of encephalopathy or seizures. Magnetic resonance imaging (MRI) at seven days showed a small amount of subdural blood, common following ventouse delivery, and a small focal left frontal lobe infarct (Figure 2). There was no evidence of hypoxic ischaemic injury or congenital brain anomaly. Guthrie testing was normal. Placental histology was unremarkable and no pathogens were isolated on maternal genital swabs.

At sixteen-week follow-up there are no developmental, behavioural, or feeding concerns. The patient has no dysmorphic features and systems examination remains unremarkable.

## 3. Discussion

The complex physiological transition from the intrauterine to extrauterine environment can be a dangerous period for the neonate. 90% complete the transition independently, with approximately 10% requiring some intervention and 1% demanding extensive resuscitation [2]. Multisystem organ adaptation to the extrauterine environment is recorded using Apgar scores, which enable clinicians to rapidly assess an infant's physiological condition and the need for medical intervention [1, 3, 4]. Typically, scores improve between one, five, and ten minutes of age [1]. There is limited literature of cases in which Apgar scores fall. Our case is novel in this regard, particularly as the patient displays no neurological sequelae.

To the best of our knowledge, there are no previously reported cases of unexplained cardiorespiratory collapse at five minutes. Multiple differential diagnoses have been considered and excluded. Sudden and unexpected postnatal collapse (SUPC) is well reported and describes infants with a normal Apgar score at five minutes who collapse unexpectedly in cardiorespiratory extremis [5]. Incidence is between 0.03 and 0.08/1000 live births and risk factors include primiparous mothers, skin to skin contact, and prone positioning [5, 6]. Given the patient's Apgar scores of eight at one minute and zero at five minutes, our case does not meet SUPC criteria.

This case provided a diagnostic challenge for the clinicians involved. Airway causes of collapse cannot explain how the patient independently established regular and spontaneous respirations at one minute of age nor explain her complete and rapid recovery. A septic screen was negative and chest imaging excluded both pneumothorax and pleural effusion. Grunting followed by rapid cardiovascular collapse due to transient tachypnoea of the newborn appears improbable given how rapidly ventilator support was weaned. There was no maternal drug history to suggest neurological and subsequent respiratory depression. The small frontal infarct is an unlikely cause of the patient's collapse. Cardiac disease is an unconvincing diagnosis in view of normal cardiac monitoring and a normal cardiovascular examination. Specific metabolic, genetic, endocrine, or neurological diagnoses are unlikely given normal Guthrie testing, rapid recovery period, and the patient's normal neurology and development at sixteen weeks. We therefore hypothesise that cardiorespiratory collapse may have occurred due to an unknown anomaly during the physiological stress of fetal-neonatal transition. Instrumental delivery was performed due to a nonreassuring CTG and one can speculate that cord gas analysis may have aided diagnosis. This case therefore acts as a reminder that although no current consensus exists regarding umbilical cord blood acid-base analysis, multiple colleges recommend that it is performed in all caesarean or instrumental deliveries, where indication is potential fetal compromise [7].

Finally, considerable literature discusses the use of Apgar scores for predicting mortality, neurological development, asphyxia, and cognitive function. Apgar scores are an accurate predictor of mortality; however, their use in the prediction of long term neurological outcome is inappropriate, particularly where encephalopathy and seizures are absent [1, 3, 4, 8, 9]. This case provides further evidence that clinicians should not rely on Apgar scores to predict neurological outcomes.

## Key Points

- To the best of our knowledge this is the first reported case of sudden, unexplained, and unexpected cardiorespiratory collapse at five minutes of age.
- This case serves as a timely reminder that cord gas analysis is recommended in all cases of potential fetal compromise and that Apgar scores should be used with caution as a predictive tool for subsequent neurological sequelae.
- Well-performed cardiopulmonary neonatal resuscitation can lead to good outcomes. It is essential that all clinicians ensure that they remain up to date with the latest resuscitation guidelines so that they have the necessary skills to perform high quality resuscitation.
